# Connexin 37 and Connexin 43 genotypes in correlation to cytokines in induced sputum and blood in cystic fibrosis (CF)

**DOI:** 10.1186/2194-7791-1-S1-A11

**Published:** 2014-09-11

**Authors:** M Ludwig, O Eickmeier, C Smaczny, F Schreiner, W Dubois, D NGampolo, R Schubert, S Zielen, R Ganschow, S Schmitt-Grohé

**Affiliations:** Abt. Allgemeine Pädiatrie, Zentrum für Kinderheilkunde des Universitätsklinikums Bonn, Germany; Institut für klinische Chemie und klinische Pharmakologie des Universitätsklinikums Bonn, Germany; Christiane Herzog CF-Zentrum des Universitätsklinikum Frankfurt, Goethe-Universität, Frankfurt, Germany

## Aims

We have provided evidence in former studies that cytokines (IL-8, TNF alpha, LBP, TGFß) measured in blood correlate negatively with lung function in deltaF508 homozygous patients. GAP junction proteins might be of importance for the influx of blood cells into the lung. Our aim was to assess the relationship between connexin genotypes and cytokines (IL-8, TNF-alpha, LBP, TGFß) in induced sputum and serum, and lung disease.

## Methods

36 patients homozygous for deltaF508 (median age 18 y, m/f 16/20, FEV_1_(%) 77) were examined. Sequence analysis was performed for genes encoding GAP junction protein alpha 1 (GJA1/connexin 43) and gap junction protein alpha 4 (GJA4/connexin 37). Cytokines were assessed in serum and induced sputum (IS) by chemiluminescence (DPC Biermann, Bad Homburg, Germany) as well as leukocyte counts.

## Results

DNA analysis was performed in 35 patients. Whereas *GJA1* showed only one rare heterozygous synonymous SNP (rs138386744) in one patient, four common SNPs were detected in *GJA4*. Two were synonymous changes, but the third variant (rs41266431) predicts an amino acid substitution (GTA → valine, ATA → isoleucine) as well as the fourth SNP (rs1764391: CCC→proline, TCC→serine). For rs41266431 patients with homozygosity for the G variant had higher IL-8 levels (median: 13.3/8.0 pg/ml, p=0.07) in serum as well as leukocytes in sputum (median: 2050/421 /µl p=0.041) than those showing heterozygosity (G/A). In individuals > 30 years lung function (FEV1 41.3/84.83 % predicted, p=0.07) was worse.

## Conclusion

SNP rs41266431 seems a promising candidate for further investigations, suggesting *GJA4* a potential disease modifying gene.

